# Experimental /numerical study of a circular rib-stiffened flange connection with inner and outer flange plates under combined bending and tensile loading

**DOI:** 10.1038/s41598-022-12896-w

**Published:** 2022-05-25

**Authors:** Yong Chen, Wending Mou, Yong Guo, Jiyang Wang, Bin Xue

**Affiliations:** 1 College of Civil Engineering and Architecture, Zhejiang University, Hangzhou, China; 2 China Energy Engineering Group Zhejiang Electric Power Design Institute Co., Ltd, Hangzhou, China; 3 State Grid Huzhou Electric Power Supply Company, Huzhou, China

**Keywords:** Civil engineering, Mechanical engineering

## Abstract

Focusing on circular rib-stiffened flange connections with inner and outer flange plates, termed inner-outer flange, the mechanical behavior of the flange subjected to the combined bending and tensile loading is experimentally studied. Four nominally identical specimens were utilized to investigate the effects of the eccentricity on the mechanical behavior. The distribution of the gap between flange plates, as well as the distribution of the bolt forces, is presented. It is found that the neutral axis would gradually approach the central axis of the flange connection, as the eccentricity increases. Moreover, provided the sufficiently strong ribs, welds, and flange plates, the capacity of the flange is found to be mainly predominated by the bolt strength. A good agreement is found in the comparison of the results obtained via the finite element analysis, the semi-analytic method (SAM) and the experimental study. It corroborates the validity of using the bolt failure assumption and the plane cross-section assumption in the SAM for approximating the capacity of the inner-outer flange. In terms of the interaction of the tensile capacity with the bending capacity, the experimental results along with those in the literature are compared with the curves defined by the codes, and suggestions for design are concluded. Yield capacity, defined as the load when the bolt stress reaches the yield strength, is recommended herein for the design of a structure under in-service condition. It is found the specifications in the current codes for the rib-stiffened flanges with a single flange plate would occasionally overestimate the yield capacity of the inner-outer flanges under the combined bending and tensile loading. Moreover, both the experimental and the numerical results show a linear load interaction curve, in terms of the ultimate capacity.

## Introduction

Circular flanges, as a bolted structural joint, are frequently employed for the connection of the tubular round-section members in tubular structures. However, the traditional circular flanges, merely possessing a single inner/outer flange plate (SI/SO flange), sometimes cannot meet the requirement for the high strength arising in the design of a tall transmission tower/pole under severe loads^1^. In view of this, provided the tubes with a large diameter, a promising circular rib-stiffened flange connection with dual flange plates, termed inner-outer flange, was developed by Deng et al.^1^, which would greatly improve the capacity of the connection, and was implemented in extensive long-span transmission tower structures, such as the 380 m tall transmission tower (Jintang tower) located in Zhoushan Islands, China^2^.

**Figure 1:**
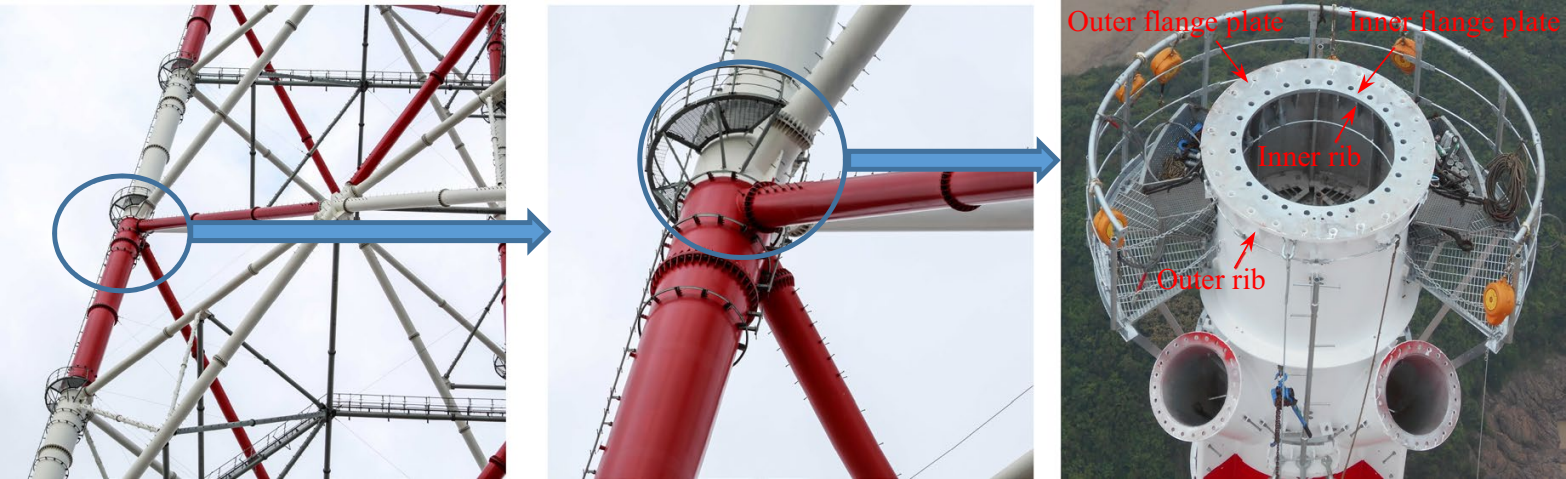
Inner-outer flange in a tubular transmission tower.

As a high-strength bolted connection, the inner-outer flanges can be identified by two main characteristics: the ribs, and the inner and outer flange plates. Figure 1 shows an actual inner-outer flange employed in a tubular transmission tower. Although the unstiffened SI/SO flange is recognized as a cost-effective connection, and widely implemented in tubular structures, the prying action^3–10^, which frequently occurs in the unstiffened flanges, would result in the increase of the bolt force, and thereby reduce the flange capacities that are mainly governed by the bolt strength. It is worth noting that various analytical models validated by experimental study^3,4^, finite element (FE) analysis^5–7^, or both^8–10^, were thus developed to accurately approximate the prying action. Stiffening the flange with ribs, as specified in both the Chinese and the Japanese codes^11,12^, is an effective method to reduce the prying action through enhancing the out-of-plane stiffness of the flange plate, and is therefore adopted in the inner-outer flanges. Note that the Chinese code entitled “Technical regulation of design for steel tubular tower structures of overhead transmission line” (DL/T 5254-2010)^11^ suggests that the prying action can be ignored for the rib-stiffened circular flanges that are normally designed. Moreover, the requirement for high capacity in connecting the round-section tubes with a large diameter (up to 2300 mm in Jintang tower^2^), and the limitation on the bolt size in the practice engineering would lead to a large number of flange bolts which cannot be well arranged in a single outer flange plate. Using an additional inner flange plate is thus proposed for the inner-outer flanges^1^, so that more flange bolts can be utilized to improve both the tensile and the flexural capacities of the flange connection.

To date, only a few studies related to inner-outer flanges were conducted. Regarding an inner-outer flange under axial tensile load, the experimental results presented by Hu et al.^13^ show that the internal tension force of the inner bolts is not equal to that of the outer bolts. A scaled-down model of an inner-outer flange in Jintang tower^2^, where the flange plates, ribs and welds were designed in accordance with the relevant guidance^11^ for stiffened SI/SO flanges, was experimentally studied by Sun et al.^14^ through a four-point bending test. It can be found that the failure mode of the inner-outer flanges is mainly the fracture of the bolts, and the neutral axis is approximately located at the distance of about 0.15 times the tube radius from the central axis (mid-axis). The bolt fracture failure was also found in the test by Xue et al.^15^, where an inner-outer flange specimen was subjected to the combined bending and tensile loads. In contrast, in the case of combination of bending and compressive loading, the experimental studies by Huang et al.^16^ and Chen et al.^17^ show that the local buckling of the tube, which commonly occurs near the flange connection, might predominate the capacity of the flange connection.

Although the design of inner-outer flanges can be completed with the aid of the numerical approaches, the absence of the relevant design guidance has greatly hindered its application to tubular structures. A semi-analytic method (SAM) was therefore proposed in the preliminary study^18^ by the authors, which is efficient in computing the ultimate capacity of the flanges under combined loads but remains complicated from the perspective of the engineering design. Moreover, the experimental evidences for the validity of the SAM, as well as the assumptions adopted, are insufficient. It deserves efforts made to perform a further investigation into the mechanical behavior of inner-outer flanges, and thereby to formulate the structural strength with an explicit form for design convenience.

In this paper, four steel inner-outer flange connections with nominally identical dimensions are utilized for experimentally study to achieve a more comprehensive understanding of the mechanical behavior of the inner-outer flanges under combined bending and tensile loading. The load–displacement curve, failure mode, development of the gap between the upper and the lower flange plates, and distribution of bolt force are obtained via the experimental approach. The experimental results are compared with the corresponding FE analysis results for crosschecking. The capacities obtained via SAM is validated by comparing them with those obtained from the FE analyses and the laboratory tests. For the inner-outer flanges under combined bending and tensile loading, a discussion on the interaction of the tensile capacity with the bending capacity is performed by comparing both the experimental results in this study and that in the relevant references with the load interaction curves defined by the current codes.

## Experimental setup

**Figure 2:**
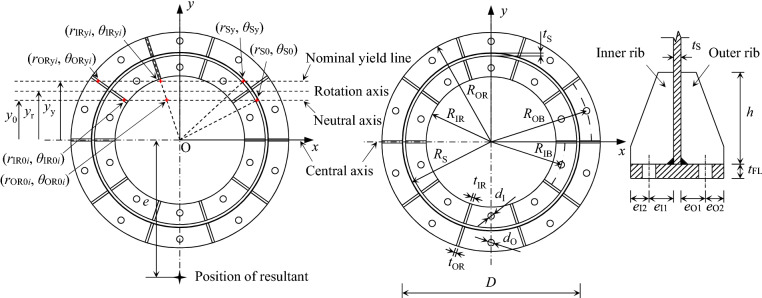
Configuration and geometric parameters of inner-outer flange connection.

Four nominally identical inner-outer flange specimens, consisting of identical upper and lower flanges, were tested. The load eccentricities for the specimens with reference numbers of T1, T2, T3 and T4 are sequentially 29.5 mm, 71.5 mm, 141.5 mm, and 961.5 mm. Figure 2 shows the configuration of the inner-outer flange, where *D* is the outer diameter of the round-section tube, *t*_S_ is the wall thickness of the tube, *t*_FL_ is the thickness of the flange plate, *e*_O1_ and *e*_O2_ are respectively the distance from the center of the outer bolt to the outer surface of the tube, and that to the edge of the outer flange plate, *e*_I1_ and *e*_I2_ are respectively the distance from the center of the inner bolt to the inner surface of the tube, and that to the edge of the inner flange plate, *h* is the height of the ribs, *t*_OR_ and *t*_IR_ are respectively the thickness of the outer ribs, and that of the inner ribs , *d*_O_ and *d*_I_ are respectively the diameter of the outer bolts, and that of the inner bolts, *n* is the number of the inner/outer bolts, and *e* is the eccentricity of the tensile load. By performing a preliminary FE analysis in advance, the specimens are carefully designed to ensure the failure of the specimens is the fracture of bolt. Table 1 lists the dimensions of the specimens. The specimens have the same total height of *H* = 1430 mm. The steel grade of the tubes, the ribs and the flange plates is Q345B^19^. The pretightening forces of the bolts are equal, namely 10.053 kN.

**Table 1:** Geometric dimensions of specimens.

*D* (mm)	*t*_S_ (mm)	*t*_FL_ (mm)	*e*_O1_ = *e*_I1_ (mm)	*e*_O2_ = *e*_I2_ (mm)	*h*/*t*_OR_ = *h*/*t*_IR_ (mm)	*d*_O_ = *d*_I_ (mm)	*n*
600	12	24	40	30	155/12	20	10

**Figure 3:**
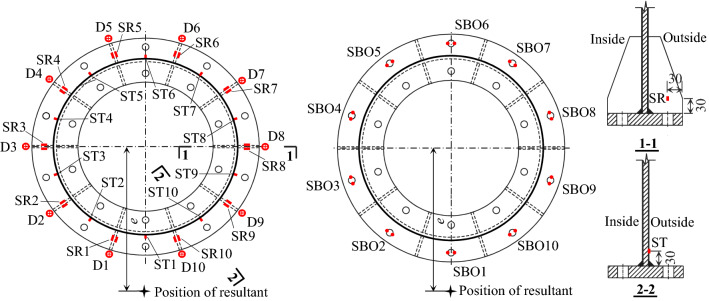
Layout of the measuring points.

The relative displacements between the outer edge of the upper flange plate and that of the lower flange plate (termed opening amount herein), the bolt forces, the strains of the tubes, and the strains of the ribs were measured. As shown in Fig. 3, the dial gauges were mounted on the edge of each outer rib to directly measure the opening amount, and the strain gages were glued symmetrically on two sides of each rib to eliminate the influence of the initial imperfections of the ribs as much as possible. The internal tension forces of the bolts were measured by using the instrumented bolts^20,21^ which were calibrated in advance. Table 2 shows the physical meaning of the capital letters in the reference numbers of the measuring points illustrated in Fig. 3.

**Table 2:** Physical meaning of capital letters in reference numbers of measuring points.

Notation	Physical meaning
D	Relative displacement between outer flange plates
SBO	Averaged longitudinal strain of outer bolt
SR	Averaged longitudinal strain of rib
ST	longitudinal strain of tube

**Figure 4:**
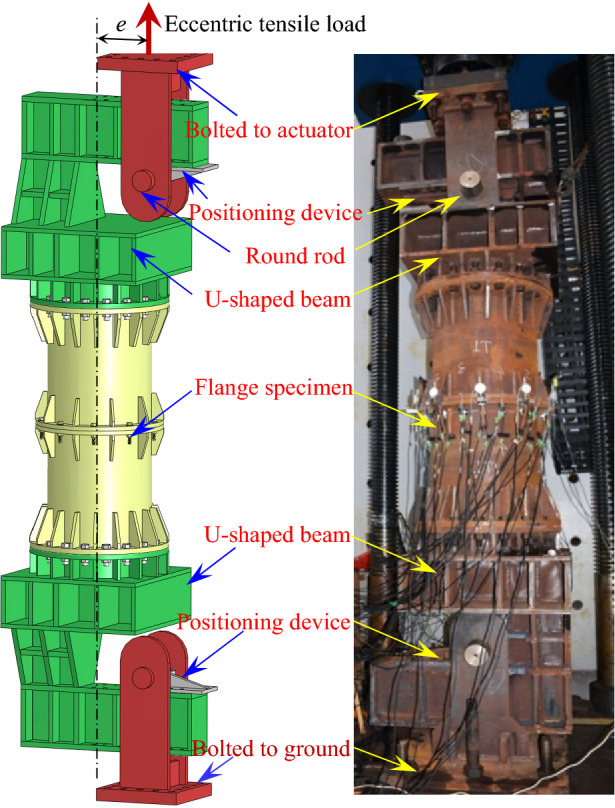
Experimental setup.

The experimental setup is shown in Fig. 4, where the axial tensile load is exerted by a microchip-controlled electrohydraulic servo multifunction test machine with a tensile loading capacity of up to 10,000 kN. As shown in Fig. 4, two identical steel U-shaped beams, possessing a relatively large flexural rigidity, are bolted to the two ends of the flange specimen respectively. The tensile load can be thereby imposed by elevating a round rod horizontally placed at distance of a prescribed eccentricity from the center of the flange. To realize a precise eccentrically loading, the round rod is horizontally constrained by a positioning device fixed to the inner bottom face of the upper U-shaped beam during the loading. Symmetrically, a round rod constrained by a positioning device is also employed and mounted at the lower U-shaped rigid beam. For each specimen, the force-controlled loading is first applied till a pronounced inelastic behavior is observed, and then the displacement-controlled loading with a speed in the range of 0.1–0.5 mm/min is imposed.

**Figure 5:**
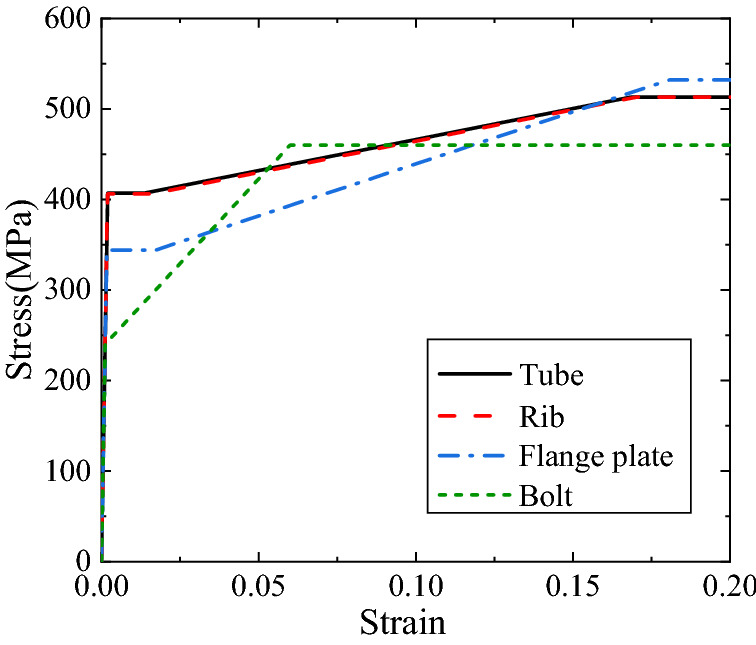
Stress–strain curves.

The stain-hardening model is employed to characterize the constitutive relationship of the materials, and is in the form of

1 $$\sigma = \left\{ {\begin{array}{ll} {E\varepsilon } & {\varepsilon < \varepsilon_{y} } \\ {E\varepsilon_{y} } & {\varepsilon_{y} \le \varepsilon < \varepsilon_{1} } \\ {E^{\prime}(\varepsilon - \varepsilon_{1} ) + E\varepsilon_{y} } & {\varepsilon_{1} \le \varepsilon < \varepsilon_{2} } \\ {E\varepsilon_{y} + E^{\prime}(\varepsilon_{2} - \varepsilon_{1} )} & {\varepsilon \ge \varepsilon_{2} } \\ \end{array} } \right.$$

where *ε* is the strain, *σ* is the stress, *E* is the elasticity modulus, *ε*_y_ is the yield strain, *ε*_1_ is the strain of yield limit, *ε*_2_ is the limit strain, and *E'* is the slope of the second slash. For bolts, *ε*_y_ = *ε*_1_. The material properties of the components, namely the tube, rib, flange plate and bolt, are the averaged values of the test results of three coupons, and are listed in Table 3 where *f*_y_ = *Eε*_y_ is the yield strength and *f*_u_ = *f*_y_ + *E'*(*ε*_2_ − *ε*_1_) is the ultimate tensile strength. The stress–strain curves of the materials of the components are depicted in Fig. 5.

**Table 3:** Material properties of components.

Components	*E* (MPa)	*ε*_y_	*f*_y_ (MPa)	*ε*_1_	*E'* (MPa)	*ε*_2_	*f*_u_ (MPa)
Tube	2.05 × 10^5^	0.00198	407	0.01355	2.30 × 10^3^	0.1684	513
Rib	2.10 × 10^5^	0.00194	406	0.01599	2.90 × 10^3^	0.1702	513
Flange plate	2.08 × 10^5^	0.00166	344	0.01718	3.74 × 10^3^	0.1807	532
Bolt	2.05 × 10^5^	0.00117	240	0.00117	3.74 × 10^4^	0.0600	460

## Numerical and theoretical approaches

### FE modelling

**Figure 6:**
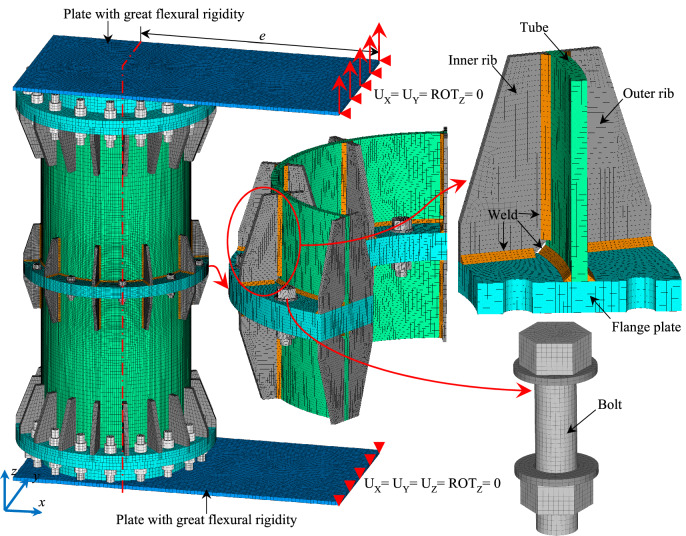
FE model of Specimen T4.

The commercial software ANSYS is employed to perform the FE analyses of the specimens. For illustration, the FE model of Specimen T4 is shown in Fig. 6. In the model, two U-shaped beams are replaced with two identical plates of great flexural rigidity (loading plate). The Young’s modulus for the loading plates is assigned to be 1.0 × 10^10^ MPa to ensure the great flexural rigidity. As shown in Fig. 6, the distance from one end of the upper loading plate to the center of flange specimen is equal to the prescribed eccentricity, therefore the eccentric load yielded by elevating the round rod is simulated through imposing identical displacements on the nodes at the end of the loading plate. At this end of the upper loading plate, the nodal degrees of freedom (DOFs) in the directions of *x*, *y*, and rotation about *z*-axis are constrained. At the corresponding end of the lower loading plate, the nodal DOFs in the directions of *x*, *y*, *z*, and rotation about *z*-axis are constrained.

**Figure 7:**
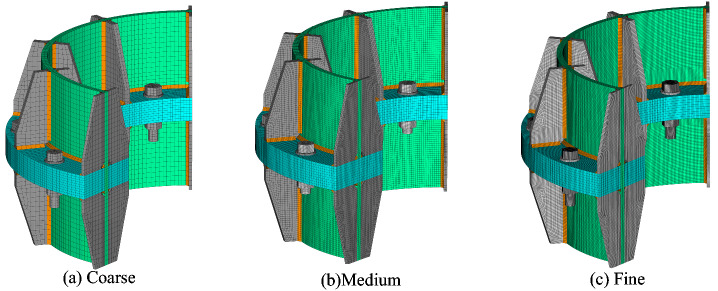
Three types of mesh.

**Figure 8:**
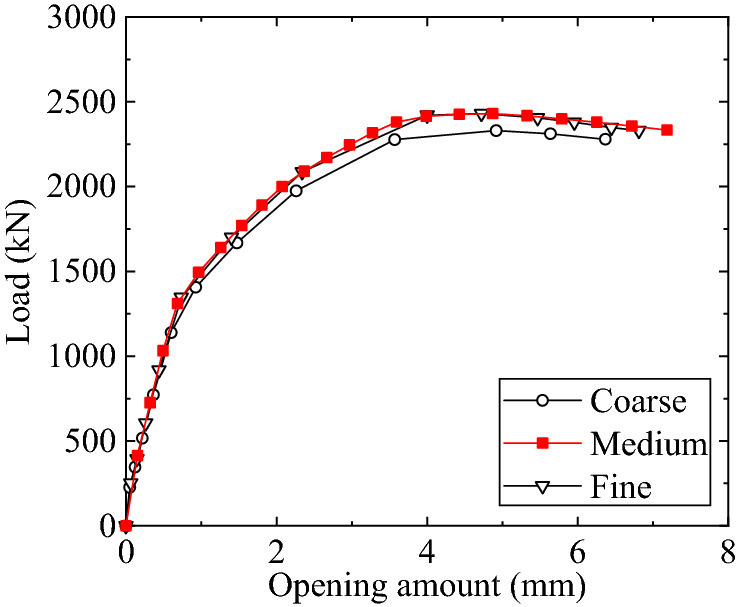
Load–displacement curves.

As shown in Fig. 6, all the components, as well as the welds with leg size of half the thickness of the tube^19^, were modelled by using the eight-node hexahedron solid elements, namely SOLID185 in the element library of ANSYS. In the preliminary study, three types of mesh shown in Fig. 7, namely coarse, medium, and fine meshes, were first surveyed. The load–displacement curves of Specimen T1 depicted in Fig. 8 show that the curve obtained by using a medium mesh almost coincides with that resulting from a fine mesh. Therefore, to balance the efficiency and the accuracy, the medium mesh is adopted herein. That is, the average mesh size of the elements in the vicinity of the flange connection is about 4 mm. The mesh size for the tube parts far from the flange is 10 mm. Since the bolts are the key component of the inner-outer flange connection, the mesh for the bolts is densified with a size of 3 mm. In addition, the mesh for the flange plate near the bolt holes is also densified. The total number of the elements is about 840,000. Pretension sections are set in the middle of bolts, and pretension loads are applied to these pretension sections to simulate the bolt pretightening forces. CONTA174 and TARGE170 elements are employed for the contact faces between the nuts and the flange plates, with the friction coefficient set to be 0.15^22^.

The preconditioned conjugate gradient (PCG) solver^23^ is employed for the static FE analysis, which is effective for the analysis of a contact problem. Both the inside and the outside bolts are preloaded with a prescribed pretightening force of 10.053 kN, which yields a normal pressure lying on the contact faces of the upper and lower flange plates, and is therefore beneficial to the convergence of the FE analysis. During the term of preloading the bolts, the method of cutback^23^ is used in the analysis to aid convergence, whereas a scheme of constant loading step is adopted in applying the external eccentric load to the flange specimens.

### Semi-analytic method^18^

**Figure 9:**
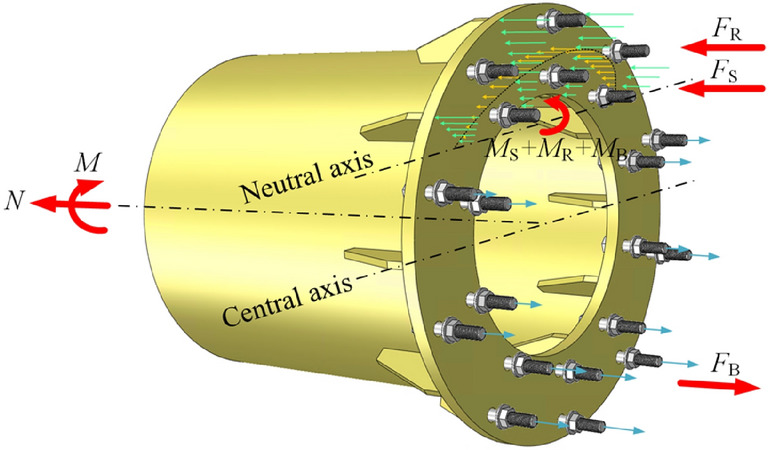
Forces on flange.

The semi-analytic method (SAM)^18^ is employed for the theoretical analysis, and restated herein for convenience. For more details, one may refer to Ref.^18^. Without loss of generality, cut the connection apart by passing an imaginary plane through the contact faces, and take one part to be studied, as shown in Fig. 9. The tube connected to the flange is subjected to the axial load *N* and the bending moment *M*, whereas the internal forces on the flange section (cut surface) are the distributed compression forces on the ribs and the tube, and the tension forces on the bolts. Note that the compressive forces originating from the flange plates between the ribs are relatively small, and are therefore ignored in SAM. Thus, the flange section can be divided into two zones by the neutral axis, namely the compression zone and the tension zone. In SAM, it is assumed that the pretightening forces of the bolts are small, resulting in a slight effect on the failure of the flange connection, and are therefore ignored. Thus, the bolts merely function in the tension zone. Cartesian coordinate system is employed with the origin set at the center of the flange section, as shown in Fig. 2. The balance of the forces yields

2 $$N = F_{{\text{B}}} + F_{{\text{S}}} + F_{{\text{R}}}$$

3 $$M{ = }M_{{\text{B}}} + M_{{\text{S}}} + M_{{\text{R}}} - Ny_{{0}}$$

where *y*_0_ is the ordinate of the neutral axis, *F* represents the resultant force in the longitudinal direction. The subscripts in Eqs. (2)–(3) and the following formulas, namely “B”, “S” and “R”, represent the variables are of the bolt, the steel tube and the rib respectively. Note that *M* in the right of Eq. (3) is the bending moment with respect to the neutral axis.

As shown in Fig. 2, a nominal yield line located at the ordinate of *y*_y_ = *ε*_y_/*φ* + *y*_0_ is employed, where *φ* is the curvature of the flange section. It is assumed that the stress of the compressive components that are above the nominal yield line would reach the yield stress. Define the rib’s symmetrical axis in the radial direction as the centerline of the rib and define the circle where the bolts are located at as the centerline of the bolts. Use the subscripts “O” and “I” to indicate the outer and inner parameters respectively. Thus, as shown in Fig. 2, *R*_OR_, *R*_IR_, *R*_S_, *R*_OB_ and *R*_IB_ are respectively the outer radius of the outer ribs, the inner radius of the inner ribs, the outer radius of the tube, the radius of the centerline of the outer bolts, and the radius of the centerline of the inner bolts. A polar coordinate system is also employed, where the polar axis coincides with the *y*-axis of the Cartesian coordinate system. Accordingly, (*r*_S0_, *θ*_S0_) and (*r*_Sy_, *θ*_Sy_) are respectively the polar coordinates of the intersections, namely the tube center line and the neutral axis, and the tube center line and the nominal yield line; the (*r*_IR0*i*_, *θ*_IR0*i*_) and (*r*_IRy*i*_, *θ*_IRy*i*_) for the *i*th inner rib, as well as the (*r*_IOR0*i*_, *θ*_OR0*i*_) and (*r*_ORy*i*_, *θ*_ORy*i*_) for the *i*th outer rib, are respectively the polar coordinates of the intersections of the rib’s center line and the neutral axis, and the rib’s center line and the nominal yield line. Note that *r*_IRy*i*_ > *r*_IR0*i*_, and *r*_ORy*i*_ > *r*_OR0*i*_. The angle between the polar axis and the center line of the *i*th outer rib, and the angle between the polar axis and the *i*th inner rib are denoted as *θ*_OR*i*_ and *θ*_IR*i*_ respectively. In addition, the center of the *i*th outer bolt and that of the *i*th inner bolt can be located by the coordinates of (*r*_OB*i*_, *θ*_OB*i*_) and (*r*_IB*i*_, *θ*_IB*i*_) respectively. Then, based on the plane cross-section assumption and the elastic perfectly-plastic model, the right terms in Eqs. (2) and (3) for a given curvature can be computed by

4 $$F_{{\text{S}}} = 2E_{{\text{S}}} \varphi t_{{\text{S}}} R_{{\text{S}}}^{2} \left[ {\sin \theta_{{{\text{S0}}}} - \sin \theta_{{{\text{Sy}}}} - \frac{{y_{{0}} }}{{R_{{\text{S}}} }}\theta_{{{\text{S0}}}} + \theta_{{{\text{Sy}}}} \cos \theta_{{{\text{Sy}}}} } \right]$$

5 $$\begin{aligned} F_{{\text{R}}} & = \varphi \sum {E_{{\text{R}}} t_{{{\text{OR}}}} \left[ {\frac{1}{2}\cos \theta_{{{\text{OR}}i}} \left( {r_{{{\text{ORy,}}i}}^{2} - r_{{{\text{OR0,}}i}}^{2} } \right) - y_{{0}} \left( {R_{{{\text{OR}}}} - r_{{{\text{OR0,}}i}} } \right) + \left( {R_{{{\text{OR}}}} - r_{{{\text{ORy,}}i}} } \right)y_{{\text{y}}} } \right]} \\ & \quad { + }\varphi \sum {E_{{\text{R}}} t_{{{\text{IR}}}} \left[ {\frac{1}{2}\cos \theta_{{{\text{IR}}i}} \left( {r_{{{\text{IRy,}}i}}^{2} - r_{{{\text{IR0,}}i}}^{2} } \right) - y_{{0}} \left( {R_{{\text{S}}} - {{t_{{\text{S}}} } \mathord{\left/ {\vphantom {{t_{{\text{S}}} } 2}} \right. \kern-\nulldelimiterspace} 2} - r_{{{\text{IR0,}}i}} } \right) + \left( {R_{{\text{S}}} - {{t_{{\text{S}}} } \mathord{\left/ {\vphantom {{t_{{\text{S}}} } 2}} \right. \kern-\nulldelimiterspace} 2} - r_{{{\text{IRy,}}i}} } \right)y_{{\text{y}}} } \right]} \\ \end{aligned}$$

6 $$F_{{\text{B}}} = E_{{\text{B}}} \varphi \beta_{{\text{O}}} R_{{\text{S}}} A_{{{\text{OB}}}} \sum\limits_{i = 1}^{{m_{1} }} {\left( {\frac{{y_{0} }}{{R_{{\text{S}}} }} - \frac{{R_{{{\text{OB}}}} }}{{R_{{\text{S}}} }}\cos \theta_{{{\text{OB}}i}} } \right)} + m_{2} f_{{{\text{yB}}}} A_{{{\text{OB}}}} + E_{{\text{B}}} \varphi \beta_{{\text{I}}} R_{{\text{S}}} A_{{{\text{IB}}}} \sum\limits_{i = 1}^{{n_{1} }} {\left( {\frac{{y_{0} }}{{R_{{\text{S}}} }} - \frac{{R_{{{\text{IB}}}} }}{{R_{{\text{S}}} }}\cos \theta_{{{\text{IB}}i}} } \right)} + n_{2} f_{{{\text{yB}}}} A_{{{\text{IB}}}}$$

7 $$\begin{aligned} M_{{\text{S}}} & = 2E_{{\text{S}}} \varphi R_{{\text{S}}}^{3} t_{{\text{S}}} \left\{ {\frac{1}{2}\left( {\theta_{{{\text{S}}0}} - \theta_{{{\text{Sy}}}} } \right) + \frac{1}{2}\left[ {\sin \left( {\theta_{{{\text{S}}0}} } \right)\cos \left( {\theta_{{{\text{S}}0}} } \right) - } \right.} \right.\left. {\sin \left( {\theta_{{{\text{Sy}}}} } \right)\cos \left( {\theta_{{{\text{Sy}}}} } \right)} \right] - 2\frac{{y_{0} }}{{R_{{\text{S}}} }}\left[ {\sin \left( {\theta_{{{\text{S}}0}} } \right) - \sin \left( {\theta_{{{\text{Sy}}}} } \right)} \right] \\ & \quad + \left. {\left( {\frac{{y_{0} }}{{R_{{\text{S}}} }}} \right)^{2} \left( {\theta_{{{\text{SO}}}} - \theta_{{{\text{Sy}}}} } \right)} \right\} + 2E_{{\text{S}}} \varphi R_{{\text{S}}}^{2} t_{{\text{S}}} \left( {y_{{\text{y}}} - y_{0} } \right)\left( {\sin \theta_{{{\text{Sy}}}} - \theta_{{{\text{Sy}}}} \frac{{y_{0} }}{{R_{{\text{S}}} }}} \right) \\ \end{aligned}$$

8 $$\begin{aligned} M_{{\text{R}}} & = \varphi \sum {E_{{\text{R}}} } t_{{{\text{OR}}}} \left\{ {r_{{{\text{ORy,}}i}} \left[ {y_{0}^{2} - r_{{{\text{ORy,}}i}} \left( {\cos \theta_{{{\text{OR}}i}} y_{0} - \frac{1}{3}r_{{{\text{ORy,}}i}} \cos \theta_{{{\text{OR}}i}}^{2} } \right)} \right] - r_{{{\text{OR}}0,i}} \left[ {y_{0}^{2} - r_{{{\text{OR}}0,i}} \left( {\cos \theta_{{{\text{OR}}i}} y_{0} - \frac{1}{3}r_{{{\text{OR}}0,i}} \cos \theta_{{{\text{OR}}i}}^{2} } \right)} \right]} \right. \\ & \quad \left. { + \left( {y_{{\text{y}}} - y_{0} } \right)\left[ {\frac{1}{2}\cos \theta_{{{\text{OR}}i}} \left( {R_{{{\text{OR}}}}^{2} - r_{{{\text{ORy,}}i}}^{2} } \right) - y_{0} \left( {R_{{{\text{OR}}}} - r_{{{\text{ORy,}}i}} } \right)} \right]} \right\} \\ & \quad { + }\varphi \sum {E_{{\text{R}}} } t_{{{\text{IR}}}} \left\{ {r_{{{\text{IRy,}}i}} \left[ {y_{0}^{2} - r_{{{\text{IRy,}}i}} \left( {\cos \theta_{{{\text{IR}}i}} y_{0} - \frac{1}{3}r_{{{\text{IRy,}}i}} \cos \theta_{{{\text{IR}}i}}^{2} } \right)} \right] - r_{{{\text{IR}}0,i}} \left[ {y_{0}^{2} - r_{{{\text{IR}}0,i}} \left( {\cos \theta_{{{\text{IR}}i}} y_{0} - \frac{1}{3}r_{{{\text{IR}}0,i}} \cos \theta_{{{\text{IR}}i}}^{2} } \right)} \right]} \right. \\ & \quad \left. { + \left( {y_{{\text{y}}} - y_{0} } \right)\left\{ {\frac{1}{2}\cos \theta_{{{\text{IR}}i}} \left[ {\left( {R_{{\text{S}}} - t_{{\text{S}}} /2} \right)^{2} - r_{{{\text{IRy,}}i}}^{2} } \right] - y_{0} \left( {R_{{\text{S}}} - t_{{\text{S}}} /2 - r_{{{\text{IRy,}}i}} } \right)} \right\}} \right\} \\ \end{aligned}$$

9 $$\begin{aligned} M_{{\text{B}}} & = E_{{\text{B}}} \varphi A_{{{\text{OB}}}} \beta_{{\text{O}}} \sum\limits_{i = 1}^{{m_{1} }} {\left( {y_{0} - R_{{{\text{OB}}}} \cos \theta_{{{\text{OB}}i}} } \right)^{2} } + f_{{y{\text{B}}}} A_{{{\text{OB}}}} \sum\limits_{j = 1}^{{m_{2} }} {\left( {y_{0} - R_{{{\text{OB}}}} \cos \theta_{{{\text{OB}}j}} } \right)} \\ & \quad { + }E_{{\text{B}}} \varphi A_{{{\text{IB}}}} 
\beta_{{\text{I}}} \sum\limits_{i = 1}^{{n_{1} }} {\left( {y_{0} - R_{{{\text{IB}}}} \cos \theta_{{{\text{IB}}i}} } \right)^{2} } + f_{{{\text{yB}}}} A_{{{\text{IB}}}} \sum\limits_{j = 1}^{{n_{2} }} {\left( {y_{0} - R_{{{\text{IB}}}} \cos \theta_{{{\text{IB}}j}} } \right)} \\ \end{aligned}$$

where *A* is the cross-sectional area, *m* and *n* are respectively the number of outer bolts and the number of inner bolts in tension zone, the subscripts “1” and “2” indicate that the bolt in the elastic state and that in the plastic state respectively. Note that in using Eqs. (5) and (8), the amount of *r*_OR0,*i*_, as well as that of *r*_ORy,*i*_, take the upper bound of *R*_OR_ and the lower bound of *R*_S_ − *t*_S_/2, namely *r*_OR0,*i*_ = *r*_OR0*i*_ if *R*_OR_ ≥ *r*_OR0*i*_ ≥ *R*_S_ − *t*_S_/2, *r*_OR0,*i*_ = *R*_S_ − *t*_S_/2 if *r*_OR0*i*_ < *R*_S_ − *t*_S_/2, and *r*_OR0,*i*_ = *R*_OR_ if *r*_OR0*i*_ > *R*_OR_. Similarly, the amount of *r*_IR0,*i*_, as well as that of *r*_IRy,*i*_, has the upper bound of *R*_S_ − *t*_S_/2 and the lower bound of *R*_IR_, namely *r*_IR0,*i*_ = *r*_IR0*i*_ if *R*_S_ − *t*_S_/2 ≥ *r*_IR0*i*_ ≥ *R*_IR_, *r*_IR0,*i*_ = *R*_IR_ if *r*_IR0*i*_ < *R*_IR_, and *r*_IR0,*i*_ = *R*_S_ − *t*_S_/2 if *r*_IR0*i*_ > *R*_S_ − *t*_S_/2.

In Eqs. (6) and (9), the coefficient *β* accounts for the inconsistency between the actual bolt strain *ε*_T_ and the nominal bolt strain *ε*_B_ = *φ*(*y*_0_–*y*_B_), namely

10 $$\varepsilon_{{\text{T}}} { = }\beta \varepsilon_{{\text{B}}}$$

**Figure 10:**
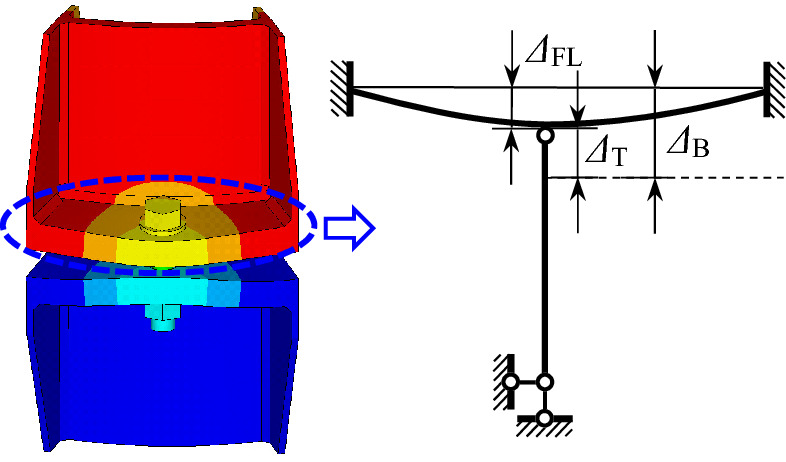
Relationship between deformations.

To approximate *β*, a simplified model, in which the in-plane deformations of the ribs are ignored, is employed and shown in Fig. 10. In the figure, *Δ*_T_ denotes the actual tension deformation of the bolt, the deflection of the flange plate is *Δ*_FL_. Thus, the nominal bolt deformation is *Δ*_B =_ *Δ*_T +_ *Δ*_FL_, which yields

11 $$\beta = \frac{{k_{{{\text{FL}}}} }}{{k_{{\text{T}}} + k_{{{\text{FL}}}} }}$$

where *k*_T_ = *EA*/*t*_FL_ accounts for the tensile stiffness of the bolt, *k*_FL_ is the out-of-plane stiffness of the flange plate. For the specimens in this paper, *k*_T_ = 2.7 × 10^6^ N/mm, *k*_FL_ = 2.3 × 10^6^ N/mm for outer flange plates, and *k*_FL_ = 3.76 × 10^6^ N/mm for inner flange plates. Thus, one obtains *β*_O_ = 0.46 and *β*_I_ = 0.58.

## Test results

**Figure 11:**
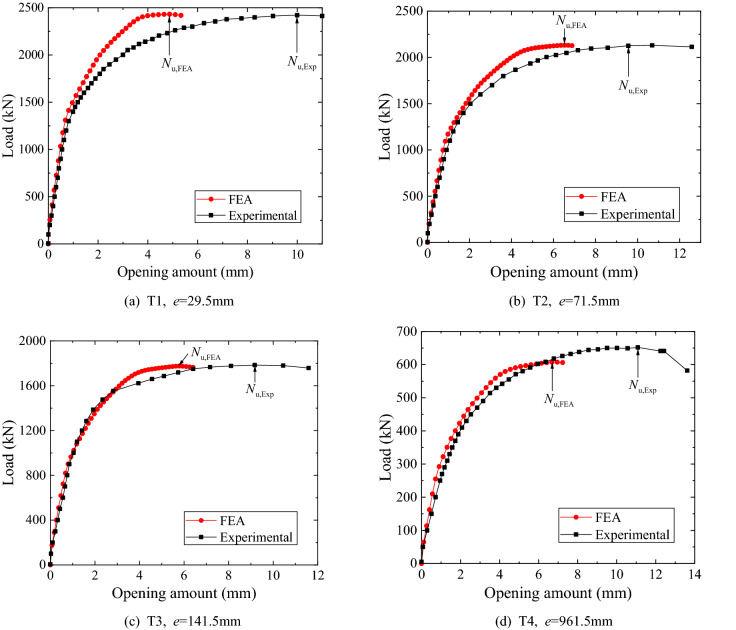
Load–displacement curves.

Figure 11 shows the load–displacement curves of the specimens, where the abscissa is the opening amount of the flange, and the ordinate is the external tensile load. The experimental results are the average of the opening amounts measured at D1 and D10 respectively. In terms of both the elastic part of the curve and the ultimate strength, the FE analysis results agree well with the experimental results. It is inferred that the discrepancy between the ultimate displacements obtained via the test and the FE analysis is largely attributed to the insufficient bolt’s limit strain employed in the FE analyses, as the post-yield opening amount is mainly predominated by the deformations of the bolts. However, a further study needs to be performed to gain insight into it, since similar discrepancies can be found in the work by Huang et al.^8^ and Couchaux et al.^9^.

**Figure 12:**
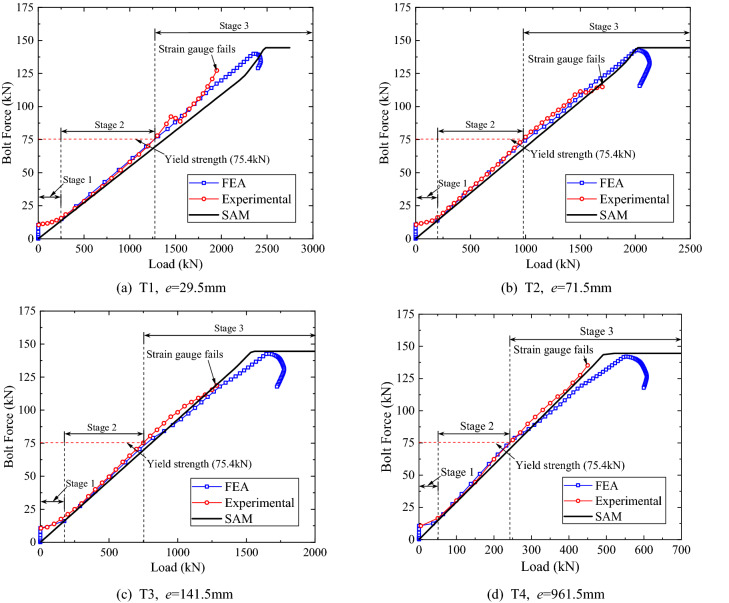
Internal tension forces of the farthest bolt (SBO-1).

Figure 12 shows the variation of the internal tension forces of the farthest bolt from the neutral axis, obtained via test, FE analysis and SAM. In FE analysis, the strain-hardening constitutive relationship shown in Fig. 5 is employed for the bolts. And, the elastic perfectly-plastic model is used for the bolts in SAM, where the yield strength of the bolt is set to be equal to the ultimate strength defined in the strain-hardening constitutive relationship for bolts. Generally, the developing of the tension force of the bolt can be divided into three stages. In the first stage, due to the pretightening force, both the FE analysis and the test results show that the upper and the lower flange plates are in contact and the bolt force is almost constant. In the second stage, namely the elastic stage, with the increase of the external eccentric load, the upper and the lower flange plates gradually separate from each other, and the bolt force increases linearly. In the third stage, the farthest bolt from the neural axis is in the elastoplastic state, and the internal forces of the other bolts would increase rapidly than before, which implies the occurrence of the redistribution of the bolt forces. In the elastic stage, both the FE analysis results and the SAM results are in good agreement with the experiment results. Moreover, the peak loads obtained via SAM are close to those obtained via FE analysis and test. It implies that the SAM is capable of capturing both the yield and the ultimate capacities, which are of primary interest from the perspective of engineering design. In addition, the comparison of the bolt forces in the specimens shows that a larger eccentricity allows the farthest bolt to yield faster, and consequently reduces the capacity of the flange.

Table 4 tabulates the yield capacities *N*_y_ and the ultimate capacities *N*_u_ of the specimens, where the subscripts “FEA”, “Exp”, “SAM” mean the values are obtained via FE analysis, experimental study, and SAM respectively. The yield capacity is defined as the load under which any bolt stress reaches the bolt yield strength (240 MPa). The ultimate capacity obtained from SAM corresponds to the bolt’s limit strain of 0.2, whereas the ultimate capacities obtained from the tests and the FE analyses are the peak values of the load–displacement curves. In SAM, the yield strength of the bolts is set to be equal to the ultimate strength in the constitutive relationship of the bolts, namely *f*_yB_ = 460 MPa. Specimen T5 in Table 4 is actually the specimen in Ref.^15^, possessing the same nominal dimensions as that of the specimens tested herein. It is found that the error of the FE analysis results is no greater than 6.8%, indicating that the FE model is capable of well predicting both the yield and the ultimate capacities of the inner-outer flanges. The yield capacities and the ultimate capacities obtained via SAM show a good agreement with the experimental results, with an error no greater than 11.2%. Generally, the SAM would a little overestimate the capacity of the inner-outer flange, which may be attributed to the use of the elastic perfectly-plastic model rather than the actual strain-hardening model.

**Table 4:** Capacities of specimens.

Specimen	*e*(mm)	Yield capacity (kN)			Ultimate capacity (kN)			Ratio			
		*N*_y,Exp_	*N*_y,FEA_	*N*_y,SAM_	*N*_u,Exp_	*N*_u,FEA_	*N*_u,SAM_	*N*_y,FEA_/*N*_y,Exp_	*N*_y,SAM_/*N*_y,Exp_	*N*_u,FEA_/*N*_u,Exp_	*N*_u,SAM_/*N*_u,Exp_
T1	29.5	1274	1271	1383	2421	2431	2622	0.998	1.085	1.004	1.083
T2	71.5	980	1014	1088	2130	2131	2346	1.035	1.111	1.001	1.102
T3	141.5	753	780	811	1784	1775	1973	1.035	1.077	0.995	1.106
T4	961.5	242	244	260	652	608	679	1.006	1.071	0.932	1.041
T5*	345.5	496**	510	531	1198**	1186	1346	1.028	1.070	0.990	1.123

*Specimen in Ref.^15^, possessing geometrical dimensions identical to the specimens in this study.

**Experimental data from Ref^15^.

**Figure 13:**
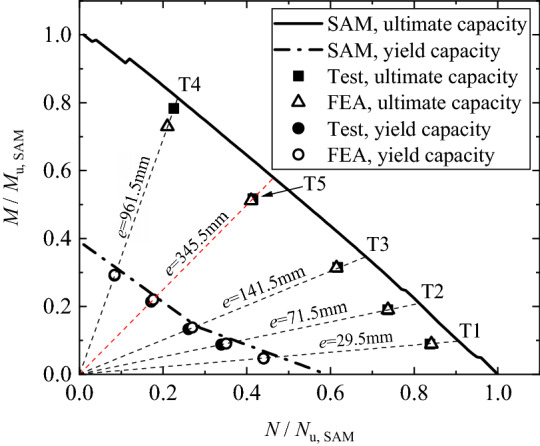
Interaction of tensile capacity with bending capacity.

Figure 13 shows the interaction of the bending strength with the tension strength, in terms of the ultimate capacity and the yield capacity. In the figure, the ultimate pure bending capacity and the ultimate pure tensile capacity obtained via SAM, i.e. the ultimate bending capacity *M*_u,SAM_ while *N* = 0 and the ultimate tensile capacity *N*_u,SAM_ while *M* = 0, are utilized to normalize the experimental and the FE analysis results. For a prescribed eccentricity, SAM is capable of obtaining the yield capacity and the ultimate capacity with consideration of proportional loading. Thus, by varying the eccentricity, the interaction curves can be obtained via SAM, whose *x*- and *y*-coordinates, i.e. the simultaneously obtained tensile and bending loads corresponding to the ultimate state, are also normalized by *N*_u,SAM_ and *M*_u,SAM_ respectively. It is found that both the experimental and the FE analysis results are slightly smaller than the corresponding interaction curve given by SAM, but show the similar trend to the curve. Generally, the interaction curve is approximately linear in terms of the ultimate capacity, and is of polyline in terms of the yield capacity.

**Figure 14:**
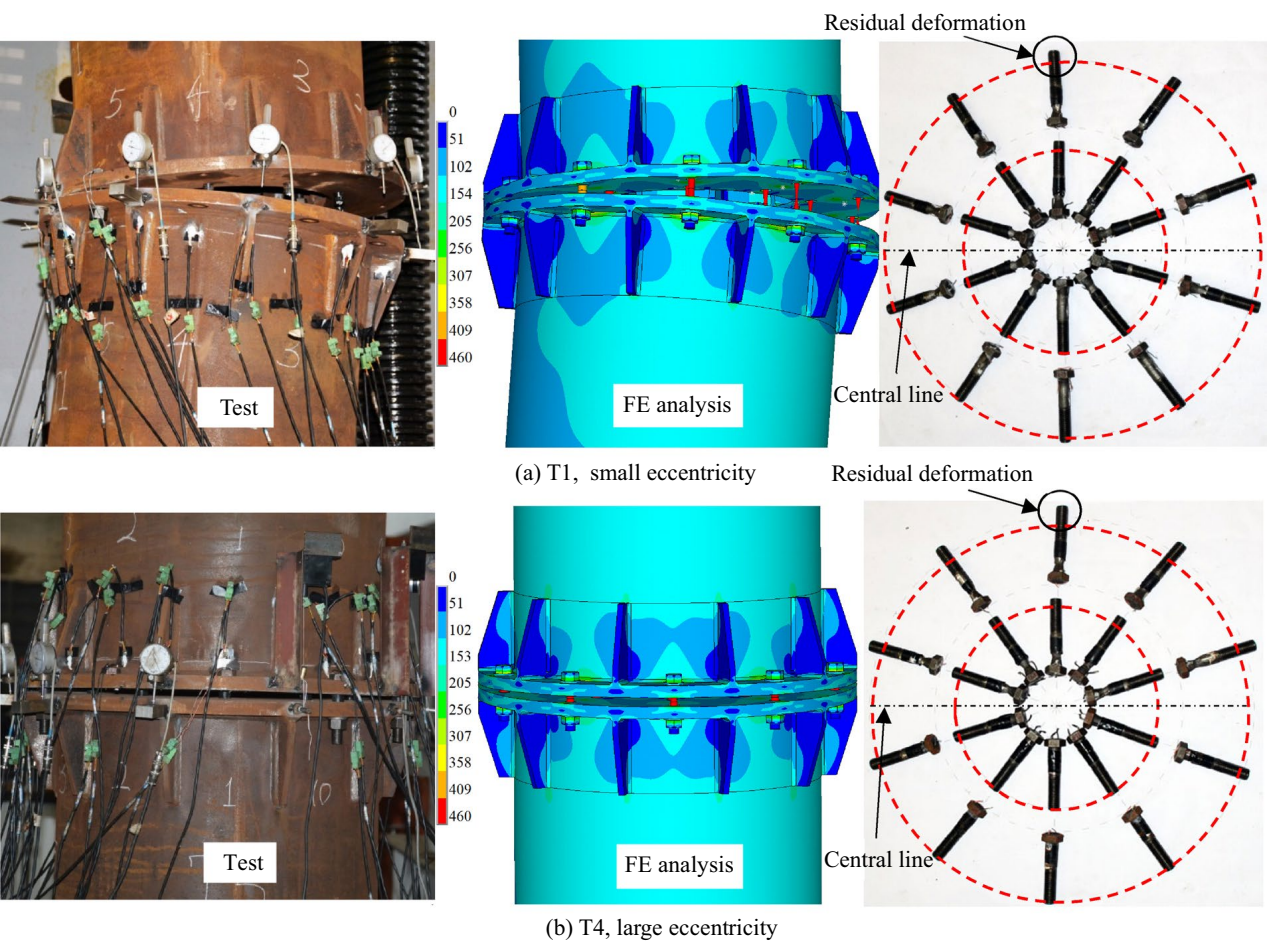
Failure modes of specimens.

In the follows, Specimen T1 (*e* = 29.5 mm, small load eccentricity case) and Specimen T4 (*e* = 961.5 mm, large eccentricity case) are taken for illustration to further show the influence of the load eccentricity. Figure 14 shows the failure modes in the cases of small and large eccentricities. It is found that the upper and the lower flange plates in tension zone are completely separated. The residual deformations of the inner and the outer bolts after failure are also illustrated in Fig. 14. The largest residual deformation, indicating the greatest tension force, is found in the farthest bolt from the neutral axis. Except for the large deformations of the bolts, neither the failure of rib nor the fracture/cracking of weld is observed in all tested specimens. Moreover, the deformations of the flange plates are relatively small while the flange fails in bearing external load. The failure of the inner-outer flange is mainly predominated by the farthest bolt from the neutral axis.

**Figure 15:**
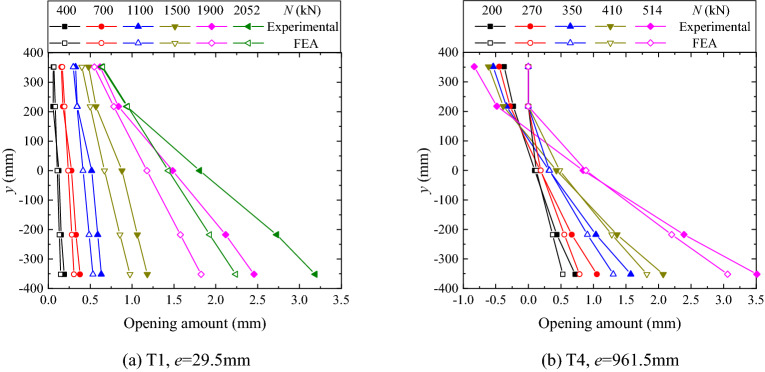
Distributions of opening amount.

**Figure 16:**
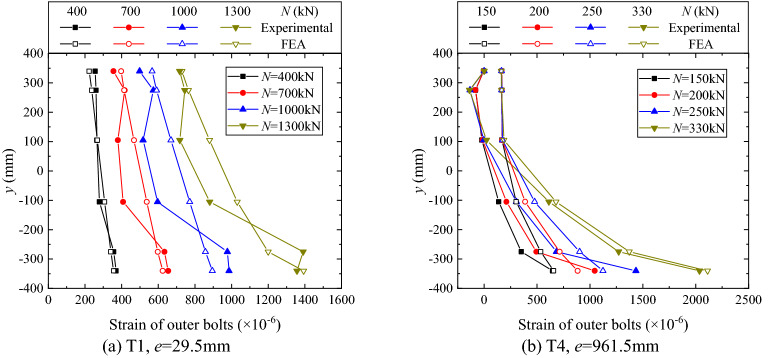
Distributions of outer bolt strain.

**Figure 17:**
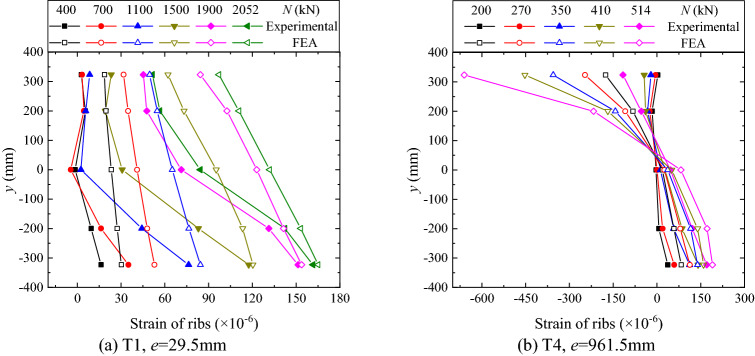
Distributions of rib strain.

**Figure 18:**
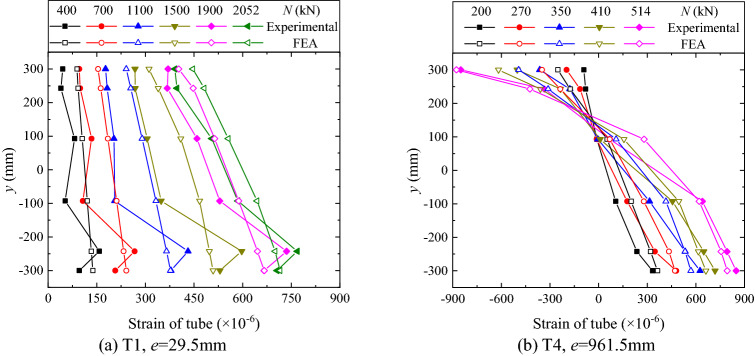
Distributions of tube strain.

The variations of opening amount with depth, under different load levels, are shown in Fig. 15. Note that the ordinates of Figs. 15, 16, 17 and 18 are the ordinate shown in Fig. 2 whose origin is set at the center of the flange. It is found in the small eccentricity case (see Fig. 15a), the variation is almost linear, whereas in the case of large eccentricity (see Fig. 15b), the nonlinearity of the variation becomes a little pronounced as the load increases. Moreover, the trend of the curves under different load levels are similar, which implies that the redistribution of the forces on the flange section is not significant. Overall, the distribution of the opening amount over the flange section largely conforms to the plane cross-section assumption, and corroborates the validity of using the assumption in SAM.

From Fig. 15, it is also found that the maximum opening amount, increasing monotonically with the increasing load, is always located on the site of the farthest outer rib from the neutral axis. As shown in Fig. 15a, in the case of the small eccentricity, both the FE analysis and the experimental results show that all the opening amounts measured are positive during the whole loading procedure, which implies that the flange plates are completely separated and the location of the neutral axis is out of the flange section. As shown in Fig. 15b, in the case of the large load eccentricity, it is found that the flange plates are partially in contact and the neutral axis is located within the flange section. That is, the neutral axis would gradually approach the central axis as the eccentricity increases. The negative values measured in the large eccentricity case may be attributed to that there is an inevitable small initial gap due to the coarse and irregular surfaces of the flange plates.

Figure 16 shows the experimental distributions of bolt strain over the flange section, along with the corresponding FE analysis results. As shown in Fig. 16a, in the case of small eccentricity, the distributions are approximately linear, and all bolts are under tensile force during the whole loading procedure. As shown in Fig. 16b, in the large eccentricity case, the compression zone where the bolt forces are close to zero can be identified. Moreover, the distributions of bolt strain over the tension zone are strongly nonlinear. These findings would be against the assumption of the linear distribution of bolt force adopted in the traditional design for a SI/SO flange^11^.

Figures 17 and 18 respectively show the distributions of the rib strain and the tube strain. As can be seen from the figures, the distributions and their evolutions are similar to those of opening amount. That is, in the small eccentricity case all the ribs and the tubes are mainly in tension and the neutral axis is identified out of the flange section, as shown in Figs. 17a and 18a. In contrast, in the large eccentricity case, the neutral axis is located in the flange section, and a flexural-type distribution of stress is identified, as shown in Figs. 17b and 18b. The difference in rib strains between the measured and the FE analysis results is relatively big. This might be attributed to the manufacturing-induced imperfection of the thin-walled rib, e.g. the unknown initial out-of-plane deformation.

## Interaction of bending capacity and tension capacity

In design of a flange connection, the required bolt forces, particularly the required maximum bolt force, depending on the external design loads, should be ascertained first, based on which the dimensions of the other flange components can be determined. For determination of the bolt forces, the so-called rotation axis method is recommended by DL/T 5254-2010^11^ and the code entitled “Technical specification for steel communication monopole” (CECS236: 2008)^24^, while the bending moment is involved. As shown in Fig. 2, the rotation axis also divides the flange section into the compress and the tension zones, and the resisting moment of the flange section are the sum of the moments of the bolt forces in the tension zone with respect to the rotation axis. Another assumption in the rotation axis method is that the bolt forces in the tension zone linearly distribute over the *y*-axis. Thus, for the SI/SO flanges under the combined bending and tensile loading, the design load interaction curve (*M*–*N* curve) is in the form of

12 $$\frac{M}{{N_{{{\text{tB}}}} \sum {Y_{i}^{2} /Y_{1} } }} + \frac{N}{{ZN_{{{\text{tB}}}} }} \le 1$$

where *N*_tB_ is the design tension strength of the bolt, *Z* is the total number of the bolts, *Y*_*i*_ is the distance from the *i*th bolt to the rotation axis, and *Y*_1_ is the distance from the farthest bolt to the rotation axis. Equation (12) can be reduced to that for a SI/SO flange under a pure bending moment (*N* = 0) or under a pure tensile load (*M* = 0), that is

13a $${\text{Under}}\;{\text{pure}}\;{\text{bending}}\;{\text{load}}:M_{{\text{C}}} = {{N_{{{\text{tB}}}} \sum {Y_{i}^{2} } } \mathord{\left/ {\vphantom {{N_{{{\text{tB}}}} \sum {Y_{i}^{2} } } {Y_{1} }}} \right. \kern-\nulldelimiterspace} {Y_{1} }},$$

13b $${\text{Under}}\;{\text{pure}}\;{\text{tensile}}\;{\text{load}}:N_{{\text{C}}} { = }ZN_{{{\text{tB}}}}$$

where *M*_C_ and *N*_C_ are the pure bending and the pure tensile capacities respectively. For SO flanges, the ordinate of the rotation axis is *y*_r_ = 0.8 *r*_S_ in DL/T 5254-2010, whereas *y*_r_ = *r*_S_ − *t*_S_ is taken in CECS236: 2008. Moreover, for SI flanges, *y*_r_ = 2*r*_S_/3 in accordance with CECS236: 2008. It is worth noting that the location of the rotation axis is still in disputation, while Eq. (13a) is applied to inner-outer flange connections.

Based on the theoretical results obtained via SAM, the load interaction curve for the inner-outer flanges under combined bending and tensile loading is concluded in the preliminary study by the author^18^, and has the form of

14a $$\frac{M}{{M_{{\text{C}}} }} + 1.556\frac{N}{{N_{{\text{C}}} }} \le 1,\quad \frac{N}{{N_{{\text{C}}} }} \le 0.45$$

14b $$1.833\frac{M}{{M_{{\text{C}}} }} + \frac{N}{{N_{{\text{C}}} }} \le 1,\quad \frac{N}{{N_{{\text{C}}} }} > 0.45$$

where *M*_C_ and *N*_C_ can be computed by Eq. (13).

**Figure 19:**
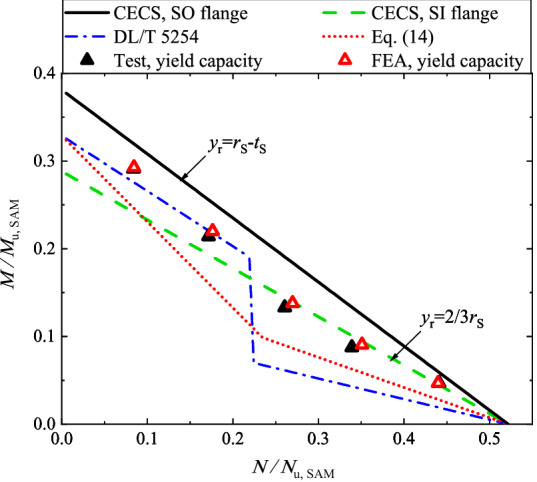
Interaction of bending capacity with tensile capacity.

In terms of yield capacity, Fig. 19 illustrates the load interaction curves defined by Eq. (14), CECS236: 2008 and DL/T 5254–2010 respectively, along with the experimental results. The curves and the experimental results are normalized by *N*_u,SAM_ and *M*_u,SAM_. In the curves, the yield strength of bolt (240 MPa) is used to determine the yield capacity. In addition, Fig. 19 shows that the experimental results are sometimes below the curves defined by the codes, resulting in a risk of failure due to the overestimation. In contrast, the curve defined by Eq. (14) seems to be conservative, as both the experimental results and the FEA results are located above the curve.

**Figure 20:**
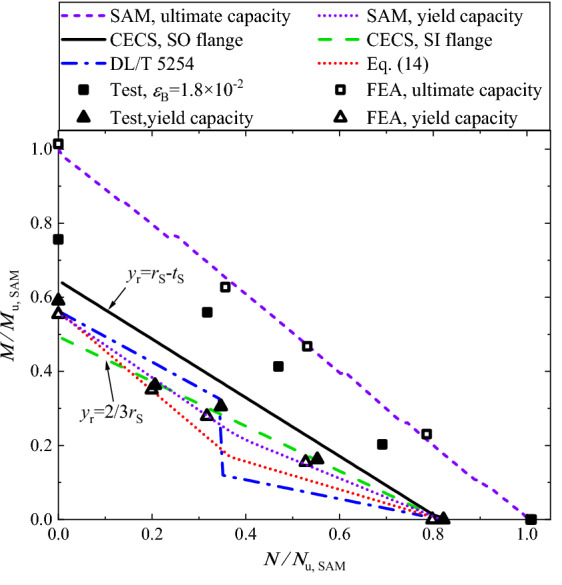
Interaction of bending capacity with tensile capacity, given specimens in Ref^25^.

Table 5 lists the geometrical dimensions of the inner-outer specimens reported by Zhang^25^, where the numbers of the inner/outer bolts are 28. Accordingly, we performed an additional study for the specimens via both FE analysis and SAM. The numerical and the theoretical capacities, along with the experimental yield capacities are presented in Table 5, which shows a satisfactory agreement. In Ref.^25^, as the tests were terminated at when a large opening amount was observed, the loads corresponding to the bolt strain of 1.8 × 10^−2^ are extracted and listed in Table 5 for reference. Figure 20 illustrates the interaction of the bending capacity with the tensile capacity, in which the results are normalized by *N*_u,SAM_ and *M*_u,SAM_ . In terms of the yield capacity, the findings are similar to that found in Fig. 19, and again highlight that the specifications in the codes for SI/SO flanges would occasionally overestimate the yield capacity of inner-outer flanges and Eq. (14) seems to be more rational.

**Table 5:** Dimensions and capacities of specimens in Ref.^25^.

Specimen	Load scheme	*e* (mm)	Dimensions (mm)				Yield capacity (kN, or kNˑm)				Ultimate capacity (kN, or kNˑm)		*ε*_B_ = 1.8 × 10^−2^ (kN, or kNˑm)	
			*D*/*t*_S_	*t*_FL_	*h*/*t*_OR_ = *h*/*t*_IR_	*d*_O_ = *d*_I_	*N*_y,FEA_	*M*_y,FEA_	*N*_y,Exp_	*M*_y,Exp_	*N*_u,FEA_	*M*_u,FEA_	*N*_Exp_	*M*_Exp_
T-A	T*	0.00	610/16	20	130/10	16	5286	0	5442	0	6671	0	6687	0
TB-A	T & B	76.25	610/16	20	130/10	16	3494	266	3659	279	5204	397	4577	349
TB-B		228.75	610/16	20	130/10	16	2030	480	2300	525	3518	805	3110	711
TB-C		457.50	610/16	20	130/10	16	1320	604	1365	625	2361	1080	2105	963
B-A	B	/	610/16	20	130/10	16	0	954	0	1017	0	1746	0	1302

*: “T” and “B” mean the tensile load and the bending moment respectively.

In terms of the ultimate capacity, both Figs. 13 and 20 show a linear load interaction curve, namely

15 $$\frac{M}{{M_{{\text{u}}} }} + \frac{N}{{N_{{\text{u}}} }} \le 1$$

where *M*_u_ and *N*_u_ are respectively the ultimate pure bending and the ultimate pure tensile capacities of the flange connection. It is worth noting that for high-rise transmission towers, the occurrence of the yield stress in the inner-outer flange bolts should be avoided, because the yield stress would result in the bolt loosening and eventually the failure of the flange joint undergoing cyclic loading such as the wind load, and the seismic excitation. Therefore, the ultimate capacity corresponding to the bolt fracture should be merely utilized to ensure the safety in the extreme load events, and the yield capacity is recommended for in-service condition.

## Conclusions

1. In terms of the yield capacity and the ultimate capacity, a good agreement between the SAM, FE analysis, and experimental results is found. Both the experimental and the FE analysis results validate the semi-analytic method. An approximately linear distribution of the opening amount over the flange section is found, which corroborates the plane cross-section assumption in SAM. Provided sufficiently strong ribs and flange plates, both the experimental and the FEA results show that the flange capacities are predominated by the bolt strength, and validate the bolt failure assumption in SAM.
2. The pretightening forces in the bolts merely affect the initial development of the bolt force, and has a negligible effect on the overall mechanical behavior of the flange. It is worth noting that the nominal bolt strain computed via the plane cross-section assumption is not equal to the actual bolt strain, due to the presence of the out-of-plane deformation of the flange plates. The relationship between the nominal bolt strain and the actual bolt strain is theoretically concluded herein.
3. The yield capacity, beyond which the bolt loosening may occur due to the plastic deformation of the bolt, corresponding to the yield strength of the farthest bolt from the neutral axis, is preferred for the design of the structures under routine operation, whereas the ultimate capacity is recommended for the design of the structures suffering the extreme load events. The experimental results, along with those in the literature, show that the load interaction curves defined by the current codes would occasionally overestimate the yield capacity. In terms of the ultimate capacity, both the experimental and the numerical results show a linear load interaction curve.
